# The Hospital. Nursing Section

**Published:** 1904-09-24

**Authors:** 


### The Hospital.

### Nursing Section.

Contributions for this Section of "The Hospital" should be addressed to the Editor, "The Hospital," Nursing Section, 28 & 29 Southampton Street, Strand, London, W.C.

No. 939.—Vol. XXXVI. *SATURDAY, SEPTEMBER 24, 1904.*

### Notes on News from the Nursing World.

### THE QUEEN OF ITALY AND ENGLISH NURSES.

The Queen of Italy, who gave birth to a son last week, previous to the event, requested the matron of Queen Charlotte's Lying-in-Hospital, London, to send her an English maternity nurse. The choice of Miss McCord fell upon Miss Margaret Brown, who was trained at Queen Charlotte's Hospital about nine years ago, and has since occupied the position of sister-midwife. She has lately been doing private nursing. This is not, however, the first occasion on which one of Queen Charlotte's nurses has been honoured with the commands of the Queen of Italy. Three years ago Miss Dickins was sent by the matron of Queen Charlotte's Hospital to nurse her Majesty when the Princess Yolanda was born, and she is still in the Royal service in charge of the two little Princesses. The fame of Queen Charlotte's Hospital has spread to all countries, and we are not surprised that her Majesty has such confidence in the members of a school whose training is the best of its kind.

### NURSES IN PORT ARTHUR.

Two statements made by Prince Radziwill, a Russian lieutenant who has just arrived at Chifu from Port Arthur, are of great interest to nurses. One is that 300 women engaged in hospital work as nurses and otherwise were advised to leave Port Arthur, but replied that they would face massacre rather than desert their post. The other is that Madame Stöessel, wife of the famous Russian general, takes the lead of the Red Cross work and is almost constant in attendance at the hospital, tenderly caring for the wounded. In the midst of this exhausting work she finds time to aid orphans and widows and superintend the making of bandages. The soldiers are said by the Prince to regard her as their guardian angel.

### QUEEN ALEXANDRA'S MILITARY NURSING SERVICE.

We are informed that Miss K. M. Bulman, Miss A. M. Orchard, Miss O. M. Griffin, Miss G. M. Allen, and Miss M. E. M. Grierson have been appointed provisionally staff nurses in Queen Alexandra's Imperial Military Nursing Service. Miss A. C. Jacob has resigned her appointment as sister.

### THE DEPRESSION IN SOUTH AFRICA.

The state of depression in the nursing world in South Africa, which a correspondent described three weeks ago, still continues without abatement. Our correspondent writes us that for the last six weeks the number of private, general, and maternity nurses out of employment in the institution with which she is connected has never for more than a day or so been less than 13 out of a total of between 50 and 60. Some of these are away, and several have lately been recommended to take hospital staff work. One nurse has had only two weeks' work in the last two months, and it is nothing uncommon for a nurse to be out one week and in two. Our correspondent herself has been exceptionally fortunate during the last six weeks, and has had five weeks' work. Each time on her return to the institute she found nurses still there whom she had left waiting for work when she went out. In one week, with 13 or 14 nurses out of employment, there were three calls only for nurses. As already intimated, this serious state of things is attributable in a measure to the general condition of affairs in the country, which makes many people unable to afford professional attendance. The special cause is the number of nurses who arrived in South Africa at the time of the war and have remained there since. Many, also, our correspondent adds, went out afterwards, attracted to the country by rumours of fortunes to be made and of an easier life than at home. In fact, they did not in the least realise that a period of depression was inevitable after the campaign; and this appears to be more acute than was anticipated even by the more far-seeing.

### NURSING IN ELEMENTARY SCHOOLS.

The Widnes Board of Education have taken an important step by appointing as one of their staff a fully-trained "Queen's" nurse, who will devote all her time to attending the schools in the town for the purpose of treating the various ills to which the children are subject, and from which they continually suffer. This is the first appointment of the kind in the North of England, though for many years the work has been done in Liverpool through the private charity of the benevolent, with the help of some of the managers. In Birmingham, too, a nurse has been visiting the schools for two or three years, under the same circumstances. In these cities the movement has the approval of the Education authorities, though as yet they do not contribute to the cost of the nurse's services. But in Widnes, thanks to the energy and enthusiasm of the clerk and some members of the board, all difficulties have been overcome, and the children in the schools under their care will have the constant care and supervision of a competent nurse. Her work is to attend to those children, class by class, as they are sent to her by the teacher, who may be suffering from sore heads, eyes, ears, toes, fingers, or sores which cause much pain if neglected, and prevent regular attendance; to note the first symptoms of any infectious disease, and thus prevent epidemics; and to send to a doctor or to a hospital any child who may have been neglected or who appears unfit to attend the school. The Widnes Board are to be congratulated upon making this forward move, and we hope that their example may be speedily followed in other towns in the country.

### THE NEW MATRON OF LEWISHAM INFIRMARY.

The appointment of Miss Henrietta Strick as matron of Lewisham Union Infirmary has given rise to a somewhat acrimonious controversy. Eleven of the guardians protested against Miss Strick's election, and addressed a letter to the Local Government Board asking that it should not be confirmed. The letter stated that the regulations of the department required that the matron of a union infirmary should have received three years' training, and that in the opinion of the signatories Miss Strick did not possess that qualification. It was alleged that she only obtained a certificate from the Leicester Infirmary for one year's training, and that she was then appointed charge-nurse at Lewisham Infirmary, a position she held without receiving any further systematic training. This letter, being submitted by the Board to the guardians for observation and information, Dr. Toogood, the medical superintendent, was asked at the last meeting of the guardians to make a verbal statement. He accordingly said that Miss Strick received two years' training at Leicester Infirmary at a time when the three years' system was not universal, and that she completed her qualification at Lewisham, where she was first staff-nurse then charge-nurse, and subsequently sister. The guardians eventually passed a resolution asking the Local Government Board to confirm Miss Strick's appointment, and the chairman mentioned that he had in his possession a document, signed by every officer in the Infirmary, expressing satisfaction that it had been made. If the Local Government Board insist on a three years' certificate, objectors to the appointment may win their case on purely technical ground; but the undoubted training and practical experience of Miss Strick fully justify her promotion.

### A NURSE AS A MEMORIAL.

Owing to the liberality of Mrs. Wilfred Marshall of Norton Manor, who has intimated her intention of subscribing £100 a year, Norton Fitzwarren and Langford are to have a parish nurse of their own. The vicar, who has been twelve years at Norton, going constantly in and out amongst the poor, has been much impressed with the want of a trained nurse in the district. A committee, including Miss Ellinor Smith, superintendent of the Somerset County Nursing Association, has been appointed to see to the carrying out of Mrs. Marshall's wishes. The nurse is provided as a memorial of the late Mr. Wilfred Marshall, who was Master of the West Somerset Foxhounds.

### NURSES' HOME AT WOODILEE ASYLUM.

Following the ceremony last week in connection with the opening of the Stobhill and District hospitals at Glasgow, at which the nurses led the singing of the Hundredth Psalm, a new nurses' home was opened at Woodilee Asylum. The chairman of the Lunacy District Board, in performing the function, said that the introduction of female nursing into the male hospital wards of the asylum had led to a great increase in the staff, and the accommodation had been for a considerable time insufficient and very unsatisfactory. He did not know any work more trying or worrying than the nursing of the insane. The dangers and abuses to which they were continually exposed were such as to try the nerves of the strongest and the temper of the sweetest of nurses, and he thought it was only reasonable that the nursing staff, after their hours of laborious service, should be comfortably housed in a building entirely apart from their work. The home contains accommodation for 110 nurses, and is as comfortable as it is commodious. The stair-case and the woodwork of the corridors are of green stained wood, the walls being in a deep shade of blue. On the ground floor there is a small and admirably equipped hospital for the use of any female attendants who happen to be ill. The kitchens are also on the ground floor and in close communication with the nurses' dining-hall. On each floor there is a housemaids' pantry, and a lift, for taking up coal and other necessaries, passes through these from top to bottom of the house.

### THE VALUE OF THE DISTRICT NURSE.

During the progress of a meeting of the Scottish Mothers' Union held last week at Tarland, Lady Aberdeen in alluding to the alleged physical deterioration of the people said that in many cases it was shown that the cause arose from the want of proper knowledge on the part of parents how to feed and take care of their children. Some of those interested in the movement for district nursing believed that there was no way in which the poor could be better helped than by having district nurses, who would assist the mothers with their children. Fears had been expressed whether this scheme were feasible in such a district as theirs, but she herself was very hopeful. Her idea was to induce a number of friends to guarantee a certain fund which would enable them to get a nurse for two or three years and cover the amount required for her salary, food, and lodging. The nurse would work under the doctor and a small committee of ladies, the nurse tending not only the sick, but the old people, if necessary. It was proposed that those who received visits should contribute according to their ability. They would then be able to judge in two or three years whether they could keep the nurse amongst them or not. Subsequently, Dr. Worcester addressed the meeting. He stated that in the establishment of district visiting nursing he would gladly spend more time and effort than in any other service on earth.

### MATRONS UNDER FORTY.

The committee of a small country hospital in Australia advertise for a matron at a salary of £45, and restrict the age of applicants to 40. The salary is very small, but the committee may possibly be so cramped in respect to funds that they cannot offer more. In these circumstances, however, it is all the more surprising that they should impose a ridiculous age limit. When a capable woman is under 40 she is often by no means disposed to settle down in a small country hospital. At 50 she views things differently, and may not be unwilling to face the prospect of spending the remainder of her working days in the superintendence of an institution which is not likely to over-tax her resources. Even in regard to activity women under 40 are not always the most energetic.

### COOKERY CERTIFICATES.

At the meeting of the Croydon Guardians last week the matron submitted a form of cookery certificate for approval, and said she would be glad to have instructions as to the arrangements to be made for the cookery classes during the coming winter. The Infirmary Committee approved of the form as suggested, and recommended that arrangements be made on the same terms as last year for a continuation of the classes in sick cookery for the nurses for the ensuing winter. This was agreed to. The form is headed "Croydon Union, Surrey," and is a certificate to the effect that the possessor has attended a course of instruction and passed a written and practical examination in sick cookery. It has to be signed by the medical superintendent, the matron, the examiner, the chairman of the Board of Guardians, and the chairman of the Infirmary Committee.

### A TRAINING SCHOOL YELL.

An American journal prints the following as "the yell of the class of 1904 at the Wichita Training School for Nurses":—

Staphylococcus, streptococcus,
Microbes all!
Sterilise and fumigate,
Watch them crawl!
Big germs, little germs,
Short and tall;
Fat germs, lean germs,
We kill them all!
Antisepsis, that's our call,
We're the largest class of all!

We are glad that English nurses do not find it necessary to yell.

### IN THE DISCHARGE OF DUTY.

It was reported at the last meeting of the Wolverhampton Guardians that one of the charge nurses in the Infirmary who had been seriously ill was now happily recovering. The illness was due largely to the devotion of the nurse to duty, and a specialist had to be called in to attend upon her. In the circumstances the Infirmary Committee recommended the payment of a fee of five guineas for his services, and the Guardians assented. It is only right that none of the cost of an illness resulting from the conscientious discharge of duty should fall upon the sufferer.

### ENTERTAINMENT TO CUMBERLAND NURSES.

On Tuesday last the Earl and Countess of Lonsdale entertained the nurses of the Cumberland County Nursing Association at Lowther Castle. Twenty-one nurses out of the 36 affiliated associations were able to be present, several being prevented coming at the last by the claims of their work. Lady Mabel Howard, hon. secretary of the County Association, Lady Audrey Buller, and others were also present, together with the retiring county superintendent, Miss Greenwood, who is taking up missionary work in India, her successor, Miss Everall, and Miss Amy Hughes, superintendent of County Associations affiliated to the Queen's Institute. The famous Lowther carriages with postilions in old-world livery, met the guests at Penrith. After luncheon the beautiful grounds were visited. It is hard to say which was most admired, the rock garden, the rose garden, the lotus garden, the Japanese garden, the unique "sweet-smelling" garden, or last but not least, the grand natural grass terrace with its wide view of fields and fells. Perfect weather added to the enjoyment of the day, which ended far too quickly, but many had to leave early in order to reach the outlying parts of the county.

### HEXHAM GUARDIANS AND THEIR HEAD NURSE.

The appointment of a head nurse at Hexham Workhouse is announced in another column. We are informed that former occupants of this post have only been on an equality with assistant nurses who have had no hospital training. The head nurse should, of course, have authority over her subordinates, and unless she has this authority it is impossible for her to discharge her duties to the satisfaction of the guardians.

### NURSES AND OUT-OF-POCKET EXPENSES.

At the last meeting of the Prestwich Guardians a letter was read from a nurse who, having been appointed to a post in the infirmary, wrote to state that she did not care to proceed with the matter. In the course of her communication, she said that she did not "take to" the hospital when she saw it; that she was not fond of nursing females; and that she would have told the Guardians at the time of her visit that she did not wish to accept the appointment, but she was afraid that if she did she would not have got her expenses paid. We do not think that the Guardians have much reason to lament the failure to enter the infirmary of a person who is not fond of nursing females; but it would conduce to straightforwardness in such cases if nurses who are invited to attend as candidates for a vacancy were paid their expenses on their arrival. In this instance the nurse excuses her lack of candour at the time of her visit by the failure of another board to even pay her train fare. While it does not follow that because a nurse is asked to appear before a board of guardians as a candidate she should consent to undertake duties which she feels convinced would be distasteful, she ought not to be expected to defray her out-of-pocket payments.

### THE ASSOCIATION FOR PROMOTING THE TRAINING OF MIDWIVES.

We understand that the office of the Association for Promoting the Training and Supply of Midwives has been removed from 12 Buckingham Street, Strand, to Dacre House, New Tothill Street, Westminster, S.W. The first two candidates trained as midwives by the Association will be ready for district work in October, and one in November.

### THE BUCKINGHAM PALACE RECEPTION.

We have received specimens of photograph post-cards representing the reception of the Pension Fund Nurses at Buckingham Palace in July, produced by the Scientific Press. Two different views show the nurses grouped in the beautiful gardens of the Palace, and we can well understand that they will be much appreciated by the friends of nurses who were present, to whom they will offer a much more vivid impression than any verbal description could convey.

### The Nursing Outlook.

"From magnanimity, all fear above;
From nobler recompense, above applause,
Which owes to man's short outlook all its charm."

### INSPECTORSHIPS.

A little while ago a municipal body advertised for a nurse as inspector, but in the end appointed a medical woman who applied, because "she alone had knowledge of public health laws."

Now, exactly as the medical schools open in October, so do the courses of lectures for women who wish to train for public work, and many of these courses should commend themselves to those who hope to become inspectors, or matrons, or take other important posts. Without some added qualification to a certificate of general nursing the chance of one of these posts is very small. And yet the work is pleasant and healthy, and an inspectorship often offers an amount of freedom that cannot be obtained in hospital work. Chief amongst the courses of lectures are those given at the Sanitary Institute in Margaret Street in preparation for the sanitary inspectors' examination. These lectures and classes being held at the Parkes Museum offer many advantages for demonstrations, and they are open to women. Even nurses who do not attend the lectures should visit the museum and see the appliances ever on view there.

Next in importance is the hygiene course at Bedford College, Baker Street. This is more thorough and more expensive; it is a nine months' course, and besides going up for the public examination, its students can also try for the special hygiene certificate given by the college. And, be it remembered, Bedford College is incorporated with London University. This is the nearest approach we have to the American course of "Hospital Economics."

The National Health Society in Berners Street generally has some good lectures in the winter, and many of its old students now hold inspectorships.

Then the Industrial Law Committee, which has its offices at Palmer Street, Westminster, though it does not prepare for examinations, yet helps to arrange for lectures, not only in London but in the provinces; it has given courses of lectures at Leeds and Manchester, for instance. And the London School of Economics in Clare Market has courses of lectures on local government, factory legislation, and so on, that are very instructive for those taking up public work.

The salary for sanitary inspectors ranges from £80 to £150; and as there is much travelling and outdoor work, it needs a strong as well as an instructed woman.

A branch of public work in which there will shortly be many openings is the new milk depôts for supplying sterilised milk; some of these are municipal—such as that at Battersea, which owes its existence to Dr. McCleary; and some private—such as that at York, which owes its existence to Mr. Seebohm Rowntree. So far special training has not been arranged in this work, but Miss Harlock (an old hygiene student of Bedford College) has been most courteous in showing and explaining the methods of the York depôt to others. And Dr. McCleary's paper in the *Journal of Hygiene* for July last is most instructive. Most nurses would be at a loss about sterilising and Pasteurising and mixing infants' milk in large quantities unless they had special training; and yet, for examining the progress of the infants, weighing them, and so on, a nurse is invaluable.

In this work, and also in posts as inspectors under the Infant Life Protection Act, a midwifery certificate is useful. But our readers know so well by now how to take their L.O.S. and get registered that we need not go into details. Some very well-paid posts as inspectors under the Midwives' Act have lately been advertised, and they are almost certain to go to those who have some experience in inspection; for theoretical teaching seldom shows how and when the inspector should undress a child to examine it, and we regret to say we have known inspectors so incompetent that they have never undressed the children, never turned up the bed to see that the mattress was clean, and have been content with a superficial examination that was positively criminal, for it gave an unwarranted sense of security. This may have been partly cowardice; in one terrible case where an inspector of boarded-out children failed of her duty because the foster-mother seemed so formidable and the outward state of the child so clean; the body of the unfortunate mite was subsequently found to be black and blue from frequent thrashings. An inspector needs to be a woman of courage and character, and also a woman of tact and sympathy The certificate of the Gynæcological Society will doubtless become of weight as it becomes better known. To have passed the Apothecaries' Hall and be able to dispense is valuable in some cases, and cookery and domestic economy certificates are occasionally useful. Massage is not so much in request as it was, and the Ling system of gymnastics and Swedish medical movements is coming more into vogue. The Board of Education's courses in hygiene and physiology are better than nothing, and at every Polytechnic some of these classes are held, and there is preparation for examination.

But on the whole the most important knowledge for an inspector is in the Public Health Acts, Factory and Workshop Acts, and the Shop Hours Act; and there is no doubt that district nurses, midwives and others should also be up in these questions, and that further arrangements should be made for teaching industrial law.

### Lectures Upon the Nursing of Mental Diseases.

By Robert Jones, M.D.Lond., B.S., F.R.C.S.Eng., M.R.C.P.Lond., Resident Physician and Superintendent of the London County Asylum, Claybury.

### LECTURE XIX.

There is a further class of mentally afflicted persons which needs special supervision and treatment. This is the large class of backward children of both sexes, the mental failure varying from slight inability to fix the attention and to learn lessons, down to the most helpless and speechless idiocy. Idiocy and imbecility include cases quite distinct in their causation and pathology, and the popular terms idiocy, imbecility, and feeble-minded indicate fairly well three degrees of mental obtuseness. The fact that such obtuseness may occur through lack of development, or through disease or injury befalling the brain, either before or after birth, justifies this as a sufficiently precise classification for our present purpose. Such feeble-minded children occur as well in the most virtuous as in the most vicious families, in the noblest as well as in the poorest and most insignificant, and when opportunities permit of education and training, suitable nurses are selected for their reasonable, methodical and affectionate discipline under which they greatly improve, and to such an extent that the slighter forms of dulness and retarded mental development evolve into reasonable, good-tempered, quiet, and obedient persons. I think, however, that it would be too much to say that feeble-mindedness is ever capable of complete cure by any amount of training; nevertheless, the results attained are eminently gratifying. To show how wide a field exists for the trained nurse for weak-minded children it will be sufficient to state that there is one weak-minded and backward child to every 120 children in the schools of London, and that every effort is made to improve such children by the application of the laws of health and elementary hygiene. This public feeling as to the improvement of the improvable cases opens up an extensive career for many nurses who are qualified to undertake such mental cases. It is important to obtain good influences over these children at an early age, and, if speech is backward, the repetition of letters, syllables, phrases, or sentences after the nurse or teacher, sometimes accompanied with music, serves as a valuable help to articulation. The discipline obtained by the repetition of nursery rhymes associated with tunes and in conjunction with rhythmical muscular movements, such as dancing, marching, drill, clapping of hands to keep time, helps to draw out their latent powers. Speech, intelligence, the use of their limbs for walking, and their hands to feed and dress themselves greatly improve under this training. The chief aim in teaching all such children is to interest them and to kindle a feeling of attachment to those around them, also to their toys, and thus serve to gain their confidence. Their hands should be guided and helped to hold things, to pick them up, to part and put things together, like a dissected map, to arrange blocks into shapes, to name, compare, and count common things, to draw conclusions and later to evolve something useful by manual application, such as is done in the various kindergarten classes of infant schools. Children who are thus being specially taught should have objects of interest, such as stuffed birds, eggs, butterflies, models of figures, pictures, simple models of machines, artificial and real flowers presented before them for observation. Due praise must be accorded to successful efforts at learning, the approval of the teacher must be used to encourage effort, the training must be pleasing and care must be taken not to fatigue or bore the child; it should also be amusing—so far as possible. The nurse will probably have much occasion to exercise her forbearance and patience in correcting uncleanly habits and in encouraging efforts at self-feeding and dressing. She must see that no one teases or scolds the child, or under any circumstances applies corporal punishment, for as such children grow older they tend to become more difficult to manage and only when the discipline is regular, kind and reasonable, will they become tractable, good tempered and quiet. With diligent application many of these cases improve so far as to support, amuse and occupy themselves. They learn a trade or a handicraft of a mechanical kind, such as tailoring or carpentering, and are in many instances self-supporting. Sometimes, when they have acquired sufficient knowledge and power to earn their maintenance, they do not know how to expend their earnings, still they learn to be of much use to their parents or relatives, especially boys who can work on the land, and girls who become useful in domestic occupations. It is of great importance to feed backward children properly and well, also to teach them to be self-helpful generally. Under suitable and judicious training even the lower grades, who tend to be noisy, mischievous, spiteful, and excitable become calm, quiet, and well behaved; uncleanly habits, tricks, and improper propensities are overcome and give way to decent and even good behaviour. Furthermore, previously weak and undeveloped physical powers are so strengthened that among the well-to-do these persons pass muster at the social meals and in the family circle without prominently intruding their deficiences and eccentricities. Also, they may be able to take part in simple pastimes such as cricket and croquet, and to become tutored, industrious, and tidy, instead of being dependent and growing up to be ungovernable, mischievous, and self-indulgent. It is interesting to note that backward children are pre-eminently susceptible to religious training and moral influences; the ceremonies and rites please them; the music during Divine service entertaining them, and the whole scene of quiet reverence usually producing placidity. After a certain age they should be encouraged to attend places of Divine worship. They learn to sit still, and, if not too long, the varied parts of the service help to fix their attention without causing weariness or fatigue. By thus fostering religious feelings a moral sense is aroused which assists to form their character, and deeds of simple kindness are quite noteworthy among these afflicted people. It is not that temptation to depravity and selfishness is not strong in this class, but it is a fact that the emotional life, such as is encouraged by religious devotions, does exercise a very considerable control over their impulsive and restless conduct. It is now no longer questionable whether these cases can be improved; on the contrary, it is generally accepted that marked benefit is derived from a distinctive system of training among the weakminded, and that much is to be obtained from persevering endeavours to develop their physical and intellectual powers; it thus becomes necessary that the mental nurse should have sound and recognised views as to how to act. The bodily condition of weak-minded children necessitates above all things good food: oatmeal porridge, and bread and milk should enter largely into the scale, and as there is so great a tendency to swallow food insufficiently masticated, it should consist also of soft puddings such as rice, custards and sago, cornflour and broths or beef tea. Solid food, such as meat, should be carefully cut up or minced, and the mince should have a due quantity of well-cooked potatoes and green vegetables, for such patients are very prone to scurvy, bad mouths, bad teeth, and sore gums; the latter often caused, in my opinion, by insufficient green vegetable food, and the daily use of a toothbrush is essential for all these cases. Fruit, such as ripe oranges, apples, etc., are greatly enjoyed, and as a rule the fruit should be cooked. Attention to the bowels is most important in all classes of mental cases. As to the clothing, it should be warm and well fitting, as the figure is often awkward and unattractive, necessitating greater attention so as to render it presentable. Outdoor exercise is very necessary to overcome the natural tendency to sloth on the one hand and to restlessness on the other. Exercise after a meal is convenient, and it is well to rest before one. Weak-minded children require more rest than is customarily taken by healthy ones; they are often unable to walk much, and they soon tire.

Of weak-minded and backward children a high proportion—it is stated over 25 per cent.—and this is verified from the statistics of Earlswood Asylum, are epileptics, and unless the fits cease there is no likelihood of improvement, rather the reverse, for progressive deterioration ensues, until the child, growing to man's estate, ultimately becomes only fit for care in an asylum for the insane. Fortunately, however, the shield has another aspect, for with combined medical supervision and judicious care on the part of the nurse, with kind training and education, also with attention to the body by means of gymnastic apparatus and easy physical drill, either in the nursery or in the playground, marked improvement often results. The epilepsy may cease, indeed, juvenile epilepsy not infrequently does, and the child grows in intellect and adds to its acquirements. It is often asked at what age is it best for the special nurse to supersede the mother? To this, with some qualifications, the answer is seven. A child at this age begins to assert and press its own will in opposition to the mother, who is often too sympathetic to deny the child anything it wants or craves for. Selfish or bad habits are evaded if proper training be commenced at this age, and if the directions of a nurse are unheeded in such cases, then treatment by association with others in a special school or institution becomes necessary; the child then sees others playing with balls, hoops, kites, or skittles, wheeling a cart or barrow, or taking an interest in live animals, and thus itself develops an interest in these by imitation and uses them to advantage without direct command. The nurse must have special training by experience, and this gives her the faith and the conviction that her influence will be a good one and that her supervision will be duly rewarded. Attention to the proper manner in which meals are taken and to the regular calls of nature are very important, care that the food may not be ravenously taken nor in too large mouthfuls, also that habits which are self-helpful are cultivated and that the children should not depend too much upon others, also that regularity and punctuality are preserved in all domestic and training arrangements. Much can be done for these weak-minded persons by gaining their confidence and then guiding them by kind and cheerful looks and words, together with tact and firmness. In the application of bodily exercises care must be taken to avoid fatigue and the nurse should carefully notice any indications of inattention or of failing health or loss of appetite. It will take much of the nurse's time to keep children who are mentally deficient clean and warm, especially in winter, as they possess languid circulations and are limited in their capability for bodily exertion. To keep the feet warm, worsted socks should be worn in bed at night. Their rooms should always be light, airy and sweet, the ventilation should be free but not draughty, the air warm but not oppressive, for which purpose open fires are preferable to the various new methods of heating by hot water apparatus or by steam radiators. The rooms should be bright and cheerful, and should have pictures and ornaments, birds or pet animals that can be looked after indoors and the nursery should not only be an indoor play-ground but also be as stated, a schoolroom full of object lessons. Care is needed in case of an outbreak of fire, and the nurse should have received instructions in anticipation of such an event so as to know how best to get her charge or charges into a place of safety. In regard to cleanliness, bathing is very necessary for weak-minded children and a tepid bath given every morning or a rapid sponging, with all the necessary precautions against taking cold, will tend to brace up and keep these little people as vigorous and healthy as is possible.

It is often found by nurses that the terminology of mental disease is puzzling and confusing, and idiocy is confounded with dementia. It is as well to understand that idiocy begins with a low grade of intelligence which gradually improves and increases, whereas in dementia the person has commenced with average intelligence which gradually diminishes. The intelligence in dementia is gradually getting less, whereas in idiocy, on the contrary, it gradually improves, and may greatly improve under skilled and patient training. In extreme cases of idiocy the stupidity is so profound that the afflicted victim lives a merely vegetative life, is totally devoid of intellect, and quite incapable of any improvement. In such a case the nurse's duty is to pay attention only to the creature or animal functions—a dull task, and one which would be positively repellent were it not for the Christian feeling that attention to the lowest and the least is a service to Him that made all things. There are many and various causes of idiocy, such as consanguineous marriages, tuberculous affections or drunkenness in the parents, hereditary and other influences, but in these the nurse is not expected to take more than an intelligent interest, and in regard to them it is not her duty to offer advice.

### The Nursing of Crippled Children.

BY AN OCCASIONAL CORRESPONDENT.

In the nursing of crippled children the sympathies of a tender-hearted woman are touched as they cannot be in any other department of nursing. Hopelessly afflicted from their earliest infancy, as many of these children are, they are naturally debarred from the advantages and pleasures of other children, and yet they display a cheerful acceptance of their limitations which it is astonishing to witness.

### Playing at "Hospital."

It is pathetic to observe them at play. What they lack in physical strength seems to be made up to them in a vivid imagination and a power of make-believe which goes far to supply many deficiencies. Playing at "Hospital" is a favourite form of recreation, and quite recently I discovered a party of little ones, with a suddenly developed eruption of large red spots upon their faces. Upon inquiry it was found that these brilliant patches were intended to represent ringworms, the colour having been abstracted from a child's paint-box conveniently near. They also bandage each other's heads and limbs, and take great pride in imitating what they have seen "Sister" do. The law of compensation makes these afflicted children find pleasure in the most

THE NURSING OF CRIPPLED CHILDREN—*Continued.*

unlikely circumstances, and for their sakes it is well that it is so.

### Skin Diseases.

There is a great tendency to skin diseases among crippled children. This can often be accounted for by a lowered vitality, a generally debilitated condition of body, and a feeble circulation. Occasionally a kind of contagious eczema attacks the scalp, the skin of which becomes soft and pulpy, and is covered with scabs; these need to be poulticed and softened with carbolic oil, and treated with whatever special ointment the doctor may prescribe. These cases are very tedious, but they do eventually respond to persevering care, though sometimes bald patches remain to show what tribulation the patient has come through. If it is a severe attack the head will require to be shaved, and frequently washed gently with soft soap. Obviously, this makes the nurse's labours easier, and as the hair soon grows again, shaving is not a very serious matter.

### Liability to Chilblains.

Ringworms, too, are very frequently to be met with on the body as well as on the head. In the former case painting with iodine is usually quite efficacious, but for the latter stronger measures have to be used. In the winter when outdoor exercise is out of the question, crippled children are greatly liable to chilblains. The nurse needs to be very vigilant in this direction, as unnoticed chilblains will develop into tiresome sores which will tax much skill and patience before they can be cured. Nourishing diet with plenty of milk, cod-liver oil, and good ventilation are indicated in these cases, sometimes massage of the limbs will prove beneficial and improve the circulation. Cripples need warm but light clothing, as their power of locomotion is naturally much less than that of normal children, and their vitality is nearly always proportionately lower.

### Hints on Important Points.

Cases which need surgical appliances also call for special attention on the part of the nurse, plaster or poro-plastic jackets are apt to chafe the skin, and the small sore spot upon an angular curvature of the spine if not promptly treated, will develop into a wound of a more or less serious nature. Irons and straps should be well-fitting and constantly inspected, surgical boots should be free from nails and from those small metal appliances beloved of some shoemakers, viz. "blakeys," which may cause a child to slip, and thereby cause serious injury. It is also necessary to see that crutches are the right length and fitted with india-rubber caps, as children grow and crutches do not, and with too-short crutches the patient will probably stoop, and further deformity will ensue. It is important that crippled children should avoid all unnecessary climbing of staircases; if possible their bedrooms should be on the ground-floor, as any extra exertion is trying and difficult for them, a fall so often leads to sad consequences. In cases of old hip-disease an accidental blow upon the site of the healed-up wound will open it up afresh, and perhaps mean long weeks of rest and treatment before it heals again.

### Chronic Constipation.

Another complication to be reckoned with among children who are crippled is chronic constipation; this, of course, is due to want of active exercise; the liver, of necessity, becomes more or less torpid, and nature must be assisted by careful dieting. Simple fruits, such as oranges, apples, stewed rhubarb, with occasional brown bread, and Quaker oats will be found very beneficial, and will generally obviate the use of aperients; in obstinate cases a simple or glycerine enema may be required. Paralysed cases need great care; they are so liable to pressure-sores; it is so easy to forget that a helpless patient when placed on a couch or an easy chair cannot change his or her position and will not be able to tell the nurse that anything is wrong. All bony prominences require constant inspection, especially elbows and shoulder-blades, and care must be taken to prevent undue contraction of limbs. Gentle rubbing will often restore the circulation in hands and feet, and will prevent or lessen the suffering which young children endure in cold weather. They should never be put to bed without some effort to warm their extremities, either in the way above indicated or by the judicious use of hot-water bottles.

### The Importance of Occupation.

Occupation for the mind and hands is very essential among cripples to prevent mental deterioration. This refers to cases which from the nature of their affliction cannot receive an ordinary education. Very often a crippled child can do something really well, and it is worth while to discover what that something is; whatever will tend to brighten the lives of these unfortunate little folks ought to be supplied. Apparently dull children have been known to develop unexpected cleverness simply through the efforts of a persevering nurse; and how greatly is one rewarded by the brightening of a dull face and the affectionate gratitude shown towards anyone who really endeavours to brighten the daily life of these afflicted children.

### Tubercular Disease.

Sometimes deformity is complicated by hereditary or acquired tubercular disease; then, of course, treatment is upon the lines of what is usually carried out in such cases, only there is very little hope of a permanent cure. Occasionally a child who has always been reckoned a helpless cripple may be encouraged to walk with crutches, and a kindly observant nurse can do much by reporting improvement to the doctor, and by patient and persevering encouragment.

### The Click-Clack of the Crutch.

No one can live amongst cripples and hear the daily click-clack of the crutch along the corridor or dining-room without feeling the keenest sympathy for these disabled and very often friendless cases; they make so little of their affliction, and are so brave and cheerful withal, that in reality they are unconsciously preaching living sermons to those of us who are strong and able-bodied and yet dare to murmur if our lot in life is not just what we would have chosen for ourselves. Surely there is much to learn from their example. Very simple things give them great pleasure. A sixpenny set of tools and a little garden of his very own spells "Paradise" to a crippled boy; he will care for it, removing all the weeds, and water it daily, and ingeniously border it round with small stones to divide it from his neighbours; while a girl is made blissfully happy with a doll and some bits of stuff to dress it with. Picture-books which allow of colouring over, scraps to be pasted in disused books, knitting stockings and scarves, bits of cloth to be made up into canvas-lined rugs—all these provide means of employment combined with enjoyment, and prevent the melancholy depression which results from a no-use-to-any-body daily life. Many people own shabby cushions, disused mailcarts, half worn-out bath-chairs, and other invalid appliances which would begin a new lease of life in a "cripples' home," and add to the sum of human happiness in no small degree, if only the one could meet the other.

### A Visit to the Aosta General Hospital.

BY AN ENGLISH HOSPITAL SISTER.

The Valley of Aosta is one of the most beautiful in Piedmont; a favoured spot truly, with its fig-trees, vineyards, and fields of maize, watered by glacier streams which come hurrying down from the snowy mountains, which surround it on three sides. Yet there is a scourge, a blight on its inhabitants. Though one can scarce imagine sickness and death here, it has been computed that one in every fifty of the population is a cretin, and that nearly half the population are afflicted with goître in some form; so Monsieur le Directeur of the General Hospital at Aosta told me. I came early one blazing July morning to see the hospital, and the doctors were still making their rounds, so the courteous director took me out into the garden, and while cutting me a bunch of gorgeous fragrant roses, told me something of the history of the hospital.

### Then and Now.

It was founded in the seventeenth century by the Order of St. Maurice, and was a very humble affair indeed in those days. It had only twelve beds, and seems to have been more used as a home for incurables than a general hospital for acute cases. Now it is a comparatively large hospital with one hundred beds, a clinique for out-patients, and a large children's ward. It is still nursed by nuns of the Order of St. Maurice, who wear soft grey dresses, white coifs, and black veils. The hospital is built round a large quadrangle, on the outside of which are three large gardens, one the sisters' garden, sweet with mignonette, jessamine, and roses; one for the patients, nicely-laid out, and the other a large well-stocked kitchen garden and orchard.

### The Staff and Wards.

Soon the doctors had finished, and we went into the wards. The staff consists of one resident and two visiting doctors, a chaplain, a head sister to each ward, with as many lay attendants as is necessary under her, varying a little with the severity of the cases. The doctors visit the wards at 9 A.M., 2 P.M., and again at 9 P.M., the morning visit being the most important. The wards are scrupulously clean and neat, the floor well-scrubbed, the walls white-washed; but they look curiously bare and austere to English eyes without flowers, plants, books, or any of the familiar ward furniture. Each bed has a white quilt and white curtains that can be drawn right round it, and a wooden locker and chair beside it. There are twenty-five beds in each ward, one side of which is devoted to surgical cases, the other to medical. Each patient has a card with his name, age, religion, and diet on it, but I saw no charts or medical notes of any kind, though there were several acute cases in the hospital just then, such as acute rheumatism and enteric fever. At the end of each ward is a beautiful little chapel with folding doors. That in one of the men's wards contained some most interesting old carving; I specially noticed a fifteenth-century prie-dieu which had been carved by the monks of St. Bernard and been lately presented to the hospital by that Order.

### The Children's Cots.

Each ward has a day-room for convalescents, and a delightful balcony where patients can be wheeled out; the children have a particularly charming one, overlooking the sisters' garden, and a magnificent view of the mountains. The children's ward has 18 cots, about 9 of which were occupied. Most of them looked happy and bright, but there were one or two extremely ill. The cots are much like our English ones, taking down at the side; the mattresses were stuffed with "zostera," which is much used in the hospital; it is a kind of dried herb—cheap, antiseptic, and non-absorbent; it is burnt after any contagious or septic case, and the mattress-cover washed and restuffed. It seems most suitable for its purpose, being soft and springy, and reminded me much of some beds I have seen in the Channel Islands used for infectious cases, which were stuffed with dried sea-weed or "vraic."

### The Operating Theatre.

Next we visited the little operating theatre. It has a terra-cotta floor, and white glazed paint walls and ceiling, and was as spotlessly clean as the rest of the hospital, which is saying a great deal. It was absolutely empty, except for a tin receiver and a brand new operating-table just received from Turin, and of which Monsieur le Directeur was inordinately proud. The instruments were kept in a cupboard outside, and I should think the College of Surgeons would rejoice to have this archaic collection for their museum of antiquities. The more important operation cases, however, are sent on to Turin, only urgent and minor operations being done here. Just under the little theatre is the clinique for out-patients; sometimes there are only five or six patients a day, but on fête days and Sundays, when the peasants come down to Aosta from their mountain homes, there are sometimes thirty or forty patients.

### Ventilation and Light.

I did not see much of the sanitary accommodation except the bath-rooms, each of which contains an ancient tin bath on wheels. The air in the wards seemed exceedingly pure, and there was excellent cross-ventilation; indeed, I did not detect an unpleasant smell anywhere, though I should think the town itself might rival Cologne with its varied stenches. Aosta is famous for hot springs, so that the hospital is well and easily heated—a great point, as the town is nearly 2,000 feet above sea-level, and the winter is long and severe. I commented on the electric light, which seemed absurdly incongruous here in the mountains, but Monsieur explained it by telling me that the rushing glacier torrents supply the motor force, so that the cost is practically nothing. And so I took my leave, laden with roses, and bringing away a very pleasant recollection of the primitive little hospital, its spotless cleanliness, its happy-looking patients, its sunny gardens, and, above all, the genial courtesy of everyone, from the director and sisters down to the little blind boy in the children's ward, who had just learnt to make his bow.

### To Nurses.

We invite contributions from any of our readers, and shall be glad to pay for "Notes on News from the Nursing World," or for articles describing nursing experiences at home or abroad dealing with any nursing question from an original point of view according to length. The minimum payment is 5s. Contributions on topical subjects are specially welcome. Notices of appointments, letters, entertainments, presentations, and deaths are not paid for, but we are always glad to receive them. All rejected manuscripts are returned in due course, and all payments for manuscripts used are made as early as possible after the beginning of each quarter.

### Everybody's Opinion.

[Correspondence on all subjects is invited, but we cannot in any way be responsible for the opinions expressed by our correspondents. No communication can be entertained if the name and address of the correspondent are not given as a guarantee of good faith, but not necessarily for publication. All correspondents should write on one side of the paper only.]

### A DELICATE QUESTION?

The Matron of the Beaconsfield Nursing Institute, 276 Balham High Road, S.W., writes: As a nurse and matron I am amused, and at the same time grieved, that such remarks should emanate from such a source, and in view of my personal experience and in justice at least to those nurses with whom I have come in contact, I must say that I have not been troubled in this way. Whether tradesmen's daughters, highly educated or otherwise, a great deal depends upon training, example, and individual character. Nurses appreciate kindness, confidence, and firmness, and where their individual comforts are considered, seldom feel inclined after their arduous duties to roam about, as stated, with either married or single men.

### AN APPEAL TO HOSPITAL NURSES.

Miss A. E. Windsor writes from Maitland Cottage, Peppard, Oxon:—May I through your paper issue an appeal to any hospital nurse with spare time to give a series of bright attractive lectures on nursing subjects—six to be given between this and Christmas at a Working Girls' Club at Old Ford on Saturday evenings between seven and nine. All travelling expenses would be paid and refreshment provided, but no other remuneration could be given. The ages of the girls vary from 13 to 18. The utmost gratitude would meet the efforts of any nurse who would undertake this kindly office and all communications should be addressed to me.

### QUALITY OF TRAINING.

"The Matron of a Cottage Hospital" writes: Having read, with interest, the article in last week's Hospital on "Quality of Training," I feel that I should like to place before you another view of the question of training in cottage hospitals. In the large general hospitals where a certificate for three years' training is given, the hospital is repaid for the trouble of training the probationer by the fact of her remaining for three years, each month of her training adding to her value as a hospital worker. If the nurse does not intend to stay for three years I think it is usually the rule that she should pay for her training, otherwise the hospital authorities are put to both trouble and expense, without any return for their expenditure—I am leaving out of the question those hospitals which repay themselves after the nurse's training by retaining her services for a fixed period as a private nurse, at a salary of, say, £30 per annum. Nurses who wish to enter a cottage hospital are probably either not strong enough for, or not within the age limits of, a recognised training school. I do not think that it would be right to bind any nurse who intends taking up nursing as a profession, to remain for three years in a cottage hospital. Therefore, I think that if a probationer wishes to make use of a cottage hospital as a preliminary training school, remaining for a year only, it is not unreasonable to demand a small premium. With no such hold on the probationer, and no "three years' certificate" in view, what is to prevent her from leaving, as soon as she has, at infinite trouble, been taught the rudiments of hospital nursing? In many cases it is both easier and quicker to do the work oneself, than to explain to a probationer how it is to be done, and to see that one's instructions have been properly carried out. In the hospital of 22 beds, of which I am matron, our staff consists of two nurses — three years' certificated — and three probationers, in addition to myself, and we do not find that number any more than we need, as we have many serious operation cases. A leading surgeon attached to a well-known London hospital, acts as our consulting surgeon, and performs the major operations. In addition, we admit any non-infectious medical cases, other than chronic. We do grant a certificate after one year's training, but this simply states the length of time which the nurse has spent here, and that she is "so far qualified to perform the ordinary duties of a nurse." On a probationer inquiring whether a three years' certificate was to be obtained here, I informed her that she could receive one for that length of time, but that it would be of no value if she wished to apply for any appointment. I do think, however, that the training here as preliminary to a larger hospital, or for any lady who wishes to gain an insight into nursing without adopting it as a profession, is more comprehensive than that to be obtained in many a larger hospital. I enclose a report for the year 1903 which will give you some idea of the nature of the cases treated here. I may mention that the committee are trying to raise funds to build a new hospital, to be worked on the lines of a general hospital, with a resident medical officer.

The Secretary of the Royal Boscombe and West Hants Hospital writes:—I have received, by your courtesy, a copy of The Hospital for September 17th, 1904, containing an article headed "The Nursing Outlook," and dealing with the training of nurses. I should not have the presumption, even had I the wish, to question the general position taken up by the writer of the article, but I cannot help thinking that, in referring to the Boscombe Hospital as an illustration of that position, a singularly unfortunate choice has been made. In the article it is implied (1) that the Boscombe Hospital is a "Cottage" Hospital, which presumably means that it has no resident house surgeon; (2) that it has no operating-theatre; (3) that a nurse might spend many years there without seeing a major operation; (4) that nothing is done, in the way of lectures, to teach the nurses their work. With regard to these points I should like to remark that:—(1) There is a resident qualified house surgeon; (2) there is an aseptic operating-theatre with complete appliances; (3) there were 40 major operations performed in 1903; (4) there are lectures given twice a week during the winter by members of the visiting staff. So far from pretending for a moment that the training at such a small hospital can, in the nature of things, be equal to that given in a large institution, I contend that anyone applying as a probationer must of necessity recognise the limitations consequent on the size of the hospital, while the certificate which she receives only mentions what, as a matter of fact, she has had in the way of training, and how she acquitted herself during that time. I have pleasure in forwarding you the annual report for 1903, and a blank certificate form, in confirmation of the above statements.

[We referred to the Royal Boscombe and West Hants Hospital as an example of a hospital with less than 30 beds which offers training for one or two years. We are glad to make it public that there are lectures for the probationers.—Ed. The Hospital.]

### Novelties for Nurses.

**By Our Shopping Correspondent.**

### A DURABLE DRESS MATERIAL.

The Nurses' Co-operative Outfitting Association, of Wellington Road, Stockport, have sent in patterns of a cotton material for nurses' dresses, called Ramine. The appearance is excellent and is obviously of exceptional durability. In order to satisfy myself that the dye is fast, I boiled a sample in strong soda water. The water was practically uncoloured and the appearance of the washed material was not changed at all. Ramine, which is a blue and white mixture, can be obtained in a number of different shades and designs. I especially recommend it for winter wear. I am sure it will make a most superior dress, an opinion I am confident that nurses will endorse if they send to the Association for patterns.

### Appointments.

[No charge is made for announcements under this head, and we are always glad to receive, and publish, appointments. The information, to insure accuracy, should be sent from the nurses themselves, and we cannot undertake to correct official announcements which may happen to be inaccurate. It is essential that in all cases the school of training should be given.]

City Isolation Hospital, Nottingham.—Miss Margaret J. Edwards has been appointed sister. She was trained at Sheffield Royal Infirmary, has been staff nurse at the Fever Hospital, St. Helen's, temporary matron of Haydock Isolation Hospital, and sister in charge at Cambridge Isolation Hospital.

Hexham Union.—Miss Rose E. Langton has been appointed head nurse. She was trained at Prescot Union Infirmary, and has since been attached to a nursing institution in Oldham.

Newport County Hospital, Monmouthshire. — Miss E. A. Brander has been appointed sister. She was trained at the Royal South Hants and Southampton Hospital. She has since been staff nurse at the Birmingham and Midland Hospital for Women, assistant matron at the Convalescent Institution, Walton-on-Thames, charge nurse at the Fountain Hospital, Tooting, London, and theatre sister at Cardiff Infirmary.

Sanatorium for Consumption at Threlkeld, near Keswick. — Miss S. Balfour-Hope has been appointed matron. She was trained at Glasgow Western Infirmary, and was subsequently matron and nurse superintendent at Kingussie Sanatorium, and matron at the Worcestershire Sanatorium, Knightwick. She has also done private nursing.

St. Mary (Islington) Infirmary. — Miss Lilian Piper has been appointed night superintendent. She was trained at Leeds General Infirmary, and has since been charge nurse at the Park Fever Hospital, Lewisham, and at Joyce Green Small-pox Hospital, Dartford.

Stephen Cottage Hospital, Dufftown.—Miss Margaret C. Fraser has been appointed matron-nurse. She was trained at the Royal Infirmary, Aberdeen. She served as a sister of the Army Nursing Service Reserve in South Africa from April 1900 to September 1902, and has since been ward sister at East Pilton Hospital, Edinburgh, and night sister in the same institution.

### Presentations.

Meltham District Nursing Association. — Miss Fillingham, who for the past seven years has acted as district nurse at Meltham, Huddersfield, has been presented by her committee with a handsome silver travelling clock prior to her departure from the district.

Padworth District Nursing Association.—Last week Miss Caroline Harford, who, for the past seven years, has been district nurse for Beenham and Padworth, was the recipient of a handsome marble clock, suitably inscribed, and a purse of 10 guineas as a token of the esteem in which she is held, and a slight appreciation of the conscientious way in which she has performed her duties. The testimonial was made up by sums varying from one halfpenny to 10s., and was subscribed to by 150 persons. Nurse Harford has since taken up her duties as district nurse for Banbury.

Parish Infirmary, Portsmouth. — On Wednesday a very pleasing ceremony took place at the Parish Infirmary, Portsmouth, when Miss J. Hall, who has held the post of matron for the past three and a-half years, was presented by Mr. Brewis, chairman of the Infirmary Committee, with various well-chosen articles on the occasion of her coming marriage. Among the presents were:— A standard lamp from the Infirmary Committee, a silver flower-stand from the medical superintendent, a silver tea-service from the resident medical officer and three other friends, a large and handsome silver tea-kettle from the nursing and domestic staff and the clerks, a silver tea-caddy and a copy of "Longfellow's Poems" from the patients, besides many other pretty and valuable gifts from former members of the nursing staff. The chairman, who eulogised the work that Miss Hall had performed during her stay in Portsmouth and spoke of the very able manner in which she had carried out her duties, said how extremely the guardians and infirmary staff generally regretted Miss Hall's departure, but mingled with their sorrow at losing her their most heartfelt wishes for every success and happiness in her future life.

### TRAVEL NOTES AND QUERIES.

By our Travel Correspondent.

Bay of St. Malo for Six Months (Holidays).—Write on a prepaid foreign letter card to Madame Pallot, Maison Mathias, St. Servan, France, and ask her if she will take you for a month at 5 francs a day. It is quite possible, as it will be the off season. You might also try Miss Goldham, Pension Primavera, Rue Ville Pepin; and Miss Humphrey, 20 Place Constantine. If they will not consent to 5 francs, it will draw forth an offer from them. At St. Enogat write to the proprietor of the Hôtel des Étrangers, St. Enôgat, France. His terms will probably be a little higher. St. Enogat is prettier than St. Servan, but not quite so well placed for excursions. Be sure to visit Le Mont St. Michel. If you want more help let me know.

November in Montreux (Maud).—Switzerland is beautiful in all seasons, but I hardly think it is the best country in which to spend November. I should rather incline to the Italian lakes. Cost about the same in both places as to living, but the expenses of the journey itself about £2 heavier on the return journey if you went to the lakes. Both situations are good for excursions. If you prefer the neighbourhood of Montreux I can tell you of a most luxuriously comfortable *pension*, above Lausanne, terms in November—about 5 fr. per day. The late autumn foliage on the Italian lakes is superb, and I think at that late season you would be better placed for visiting the adjacent towns. You have some time before you so let me hear again what you think of the relative merits of North Italy and Switzerland. Tremezzo, Cadenabbia, or Bellagio are places I should recommend; they are all on Lake Como and therefore very easily reached.

Second Answer about Bay of St. Malo (Holidays).—I do not think you will find any accommodation in Dinard itself at your terms. Try Madame Savina, Villa Michelet, Boulevard St. Enogat; St. Servan is also on the sea and St. Enogat only half a mile from it. You reach Dinard *via* St. Malo, direct from Southampton; second class return, £2 1s. The length of time on the journey varies according to weather; average time, 10 hours. On arriving at St. Malo five minutes' walk from the custom house takes you to the little Dinard steamer.

Rules in Regard to Correspondence for this Section.— All questioners must use a pseudonym for publication, but the communication must also bear the writer's own name and address as well, which will be regarded as confidential. All such communications to be addressed "Travel Correspondent, 'Nursing Section of *The Hospital*,' 28 Southampton Street, Strand." No charge will be made for inserting and answering questions in the inquiry column, and all will be answered in rotation as space permits. If an answer by letter is required, a stamped and addressed envelope must be enclosed, together with 2s. 6d., which fee will be devoted to the objects of "The Hospital" Convalescent Fund. Ten days must be allowed before an answer can be published.

### Echoes from the Outside World.

### Movements of Royalty.

The King attended Crathie Parish Church on Sunday morning, and afterwards the Prince and Princess of Wales, who with their children were also present at the service, were invited to luncheon with his Majesty at Balmoral. The Grand Duke Michael was also one of the guests. In the afternoon the King visited the Duke and Duchess of Fife at Mar Lodge, and in the evening the Very Rev. Dr. Gillespie and the Rev. S. J. Ramsay Sibbald, minister of Crathie, dined with him. On Monday the King left Balmoral for Glenquoich on a visit to Lord and Lady Burton. The Queen, with Princess Victoria, arrived at Copenhagen on Sunday, and was met by the King of Denmark and many other members of the Danish Royal Family. The King of the Hellenes was one of the guests at the dinner at Bernstorff Castle, where the Queen is staying. The Queen on Monday visited the Crown Prince and Princess at Charlottenhind Castle, and Princess Victoria went for a bicycle ride with two of the Danish Princes.

### The Heir to the Italian Throne.

The birth of an heir to the Italian Throne took place on Thursday night last week at the Castle of Racconigi, near Turin. The event was proclaimed to the inhabitants by a salute of 101 guns, and the bell of the Capitol was rung. The Heir Apparent has been named Humbert, and will bear the title of Prince of Piedmont. King Victor Emmanuel III., then Crown Prince, was married at Rome on October 24th, 1896, to the Princess Elena of Montenegro, and their first child, the Princess Yolanda, was born on June 1st, 1901, her sister, the Princess Mafalda, being born on November 19th, 1902. All the European Sovereigns telegraphed their warmest congratulations to the King and Queen. The King has signed decrees of amnesty and indulgence for crimes not wilfully committed, for press offences, for desertion by sailors of the merchant marine, and for all minor contraventions of the law, also an amnesty for crimes committed in the colony of Eritrea before January 1st, 1897. His Majesty has telegraphed to the Prime Minister, Signor Giolitti, informing him that he has contributed a million lire—£40,000—to the workmen's old age pension fund.

### The Siege of Port Arthur.

Some extraordinary statements by Prince Radziwill, a Russian lieutenant who has just arrived at Chifu from Port Arthur, whither he went with dispatches to General Kuropatkin, have caused considerable sensation. He says that now the combatants on both sides are absolutely venemous in the fury of their antagonism. During the assaults of the last four days of August two companies of Japanese, finding themselves at the mercy of the Russians, hoisted the white flag. Of this the Russians took no notice, but fired volley after volley, while the Japanese in the rear expressed their disapproval of the intended surrender by firing into their comrades. Caught between two fires, both companies were annihilated, 600 men being shot down as they stood. For days afterwards, wounded men were seen lifting their arms and fluttering handkerchiefs in impotent appeals for help, but the Russians were afraid to venture out. The Prince himself saw in the midst of the heaps of dead a Russian and a Japanese lying locked in a death embrace, the Japanese with his teeth sunk in the Russian's throat, while two of the Russian's fingers were buried in the eye sockets of his foe. In consequence of the non-observance of flags of truce, the Japanese dead are, in many cases, still unburied, and the stench is so bad that the soldiers plug their noses with wadding soaked in camphor.

### Death of Prince Herbert Bismarck.

The death of Prince Herbert Bismarck, who had been unconscious for some days, took place at Friedrichsruhe on Sunday morning. The telegrams of condolence included messages from King Edward and the German Emperor. Prince Herbert was the eldest son of the famous Chancellor of the German Empire, and was in his 55th year. He served in the war of 1870, and was badly wounded. In 1876 he passed the necessary examination for the Diplomatic Service, and in 1882 was attached to the German Embassy in London as Councillor. He had several friends in England. From 1881 to 1886 he was a member of the Reichstag, but in 1890, on his father's retirement, he also tendered his resignation, returning in 1893 to politics.

### The Late Bishop of Carlisle,

The remains of Dr. Bardsley, Bishop of Carlisle, who died last week, were laid to rest on Saturday. In accordance with the bishop's express wish, the service was of the most simple character. He had sent from his death-bed a message to his clergy that he did not wish any of them to feel under any obligation to attend his funeral at personal inconvenience to themselves. A large gathering of clergymen, from all parts of the diocese, nevertheless attended. Dr. Bardsley, who was in his 69th year, was Bishop of Sodor and Man from 1887 to 1891. He was one of the few cycling prelates, and with 300 churches spread over a vast area, he found the bicycle of great use to him.

### A Record Walk.

The vegetarians, total abstainers, and non-smokers are rejoicing greatly over the recent success obtained by Mr. Geo. Allen, who has broken the record for time for a walk from Land's End to John o' Groats, a distance of close upon 1,000 miles. The previous record was 24 days 4 hours, whereas Allen has accomplished the distance in seven days less. The pedestrian not only increased his mileage every day, but also his speed. The second week he walked more than 48 miles further than the first week, and during the last five days considerably further than he did during the first six days, and nearly as far as in the whole of the second week. During the last two days he covered an average of 88½ miles per day, and this after having walked 900 miles.

### "The Tempest" at His Majesty's Theatre.

The latest production by Mr. Tree at His Majesty's Theatre bids fair to rank highly amongst his list of successes. "The Tempest" is magnificently staged, and the air of enchantment and witchcraft with which Shakespeare invested his play is cleverly preserved. Amongst the most striking scenes is the first, when, instead of a conversation about a shipwreck which was originally the only way of representing the disaster, not a word is spoken, and we have a thoroughly realistic picture of a wreck, thunder and lighting, real water and real wind, and the poor vessel, her masts having gone by the board, breaks up before the eyes of the spectators. The yellow sands also make a beautiful scene, and the ballet between naiads and reapers who are taught love by a delightfully impudent little Cupid, who afterwards acts as clergyman to marry the couples, introduces a pleasant interlude. As to the acting, the character of Ariel has a charming representative in the person of Miss Viola Tree. Mr. Tree himself is sufficiently repulsive as the monster whose powers of speech have been educated, it is true, but who in all other respects remains a savage beast. Miss Norah Kerin acts creditably, but is a trifle too studied in manner; Mr. Basil Gill looks handsome; Mr. Lionel Brough and Mr. Louis Calvert supply drunken drollery in truly Shakesperian style.

### A Book and its Story.

A FIFTEENTH CENTURY ROMANCE.*

Miss Hawtrey's quaint and tragic romance "Perronelle" is distinctly attractive. She has caught the atmosphere of mediæval Parisian life exactly, and she writes with an ease of its manners and customs possible only to one who has studied the period intelligently and sympathetically. Perronelle Duchesne is a very human study of a wilful, high-spirited girl of the French middle-class who is the victim of an arranged marriage. Her first view of her future husband is at the church-door on the wedding morning. Poor little Perronelle! At once the reader's sympathy is aroused by the vision of the mere child who is to be offered on the altar of material sacrifice to a sour-visaged bridegroom double her age. But the spirit of revolt, if temporarily subdued, is as active in Perronelle as in any twentieth-century maiden. "Forgetting her mother's maxims for the conduct of young wives, she met Maître Gilles' eyes with a half-timid, half-sullen scrutiny. What sort of a man might this be? She thought him ugly and she regarded him with repulsion and fear, and a vague feeling of rebellion. She had been handed over to him as a piece of furniture for his house; no choice had been offered her; no appeal against his authority. But his temper was unknown to her, and the mere fact that she knew nothing of his nature, stimulated her with a reckless curiosity." Perronelle herself is standing at the door of a room in her new home, waiting for Maître Gilles to address her. "Her white coiffe was simply arranged to conceal her hair, but two little auburn curls upon her temples and another at the nape of her neck had escaped from beneath its folds. . . . Her large, well-set grey eyes were over direct in their gaze to suit fifteenth-century ideas of propriety." To her elderly husband this look of penetrating scrutiny was particularly disconcerting. It was so contrary to all preconceived notions of feminine modesty. "Maître Gilles disapproved of her straight look. . . . For a moment there was silence. Then he cleared his throat, and, laying down his pen, he folded his arms and began to speak slowly, weighing his words. "Perronelle!" "Yes, sir," said Perronelle. "You are young and inexperienced. Young and inexperienced," he repeated emphatically. "You are entering upon a new state of life. Doubtless you will make mistakes; doubtless, I say, in spite of the training you have received from Dame Agnes, your worthy mother, you will make mistakes." "I suppose so," said Perronelle, apprehensively. "However, if you will show yourself penitent and humble-minded, and anxious to amend your errors, and to learn to accomplish my pleasures, I am willing to overlook your first failures. I will overlook them." He paused. "If you are in any perplexity, Dame Berthine will assist you with counsel. She is an experienced housewife, and she will take charge of the maids, while Maître Jahan will superintend the men. But you must remember that you, as my wife, are mistress of the household; you are responsible," said Maître Gilles, well pleased with the homily he had prepared. "Yes, sir," said Perronelle. This interview had taken place soon after the marriage. Perronelle liked nothing about Maître Gilles. His voice, which was harsh and thin in tone, jarred on her ear; he was sordid and mean by nature, and on this particular morning was casting up the expenses of the wedding feast. He was a rich man and did not require money with his wife. He had chosen Perronelle because she came of a good stock and her mother had borne the reverses which had followed the loss of her husband with fortitude. But in spite of her home-training, Perronelle's nature was one which required a congenial setting to bring out its better side, and her present circumstances were in every way antagonistic to this. She loved beauty in every form, she had an innate craving for fine clothes and the hundred-and-one things that every woman who is pretty and artistic likes to have around her. Maître Gilles did not stint her allowance, and the possession of money of her own to spend was a new and enchanting experience. When she had left Maître Gilles to his calculations, after asking permission to go out, which was granted on condition only that she was accompanied by Dame Berthine, she found herself in a large hall from which an open door led into a courtyard. Between Perronnelle and the world outside stood a massive door which was barred. "The hall was hung with tapestry representing the patient Griselda, but save for long benches placed against the walls it was empty. The sun streamed in from the garden and was shining upon the further side of the court, where pigeons, with irridescent necks, were pecking grain, strutting and cooing and trimming their feathers, and a peacock was spreading his tail in silent satisfaction." From the hall a staircase led to a narrow gallery. She longed to be out of doors, and yet, while there remained unexplored corners of her new home, these must be first inspected. Two closed doors faced her. "There is plenty of time," she thought; and since there was no one to bid her be more staid, she ran across the hall, her skirts trailing the fresh straw with which the floor was covered. She opened one of the doors and entered a room. The walls were hung with blue damask and scented with spices. Several great carved lecterns supported open books; a few stools and stiff-backed chairs, carved and cushioned with dull blue stuff, and one or two round book tables, whereon lay massive parchment volumes closed and clasped, completed the furniture of the room. Perronelle wandered from one lectern to another. She examined the titles and found the books were either chronicles or religious treatises. These had no attraction for her. But her eye caught the name of one other volume. "Le Romaunt des Deux Amants." She had difficulty in deciphering the curious characters, delicate though the small square writing was in which the mediæval romance was set forth. But she read enough of it to turn away with tears in her eyes, her cheeks flushed, and a wistful longing in her heart. Where was the prince for which she waited? The sound of a step on the stair and the rustle of a skirt startled Perronelle; she shut the book and went quickly into the hall where she met Dame Berthine, who opened her lips to address her, but Perronelle hastened to speak first. "I want to go out," she said, "and you must come with me. He said so." She nodded towards the closed parlour door. Dame Berthine remonstrated in vain, reminded her young mistress of household matters. "But," said Dame Berthine, "there is so much to be done; the linen is not yet in the presses and the maids are new." . . . "I am going out," said Perronelle obstinately. Once outside the house Perronelle, enchanted by the crowd and the gay scene, gazed about her in a way that called forth frequent reproofs from her duenna. Suddenly there was a sound of shouting; they went forward and saw a scrambling crowd of beggars hunting for pence in the mud. In their midst rode Louis, Duke of Orleans, handsome, scornful, magnificent in blue and silver, escorted by two or three esquires. "Perronnelle!" whispered Dame Berthine, scandalised, "what are you doing?" For Perronelle was eagerly gazing up at the Prince . . . and as he rode past his keen glance singled her out from the rest. For that glance Perronelle pays dearly, and the rest of the story is concerned with its result which causes her much suffering and life-long expiation.

* "Perronelle." By Valentina Hawtrey. John Lane. 6s.

### Notes and Queries.

**FOR REGULATIONS SEE PAGE 143.**

*Instrument Makers.*

(188) Will you kindly give me the address of manufacturers of rubber appliances and instrument makers ?—*Mrs. W.*

The names of good instrument makers, etc., are advertised in our columns weekly. We cannot recommend individual firms.

*Free Home.*

(189) Can you mention the name of any free home to which an epileptic girl of 27 could be sent ?—*Pater.*

We do not know of any free homes, but the Poor-law authorities provide for persons who cannot pay.

*Australian Army Nurses.*

(190) Can you inform me if Australian Army nursing sisters can be transferred to the Queen Alexandra's Imperial Military Nursing Service ?—*Sister M.*

Appointments to Queen Alexandra's Imperial Military Nursing Service are only made in accordance with the conditions laid down in the regulations, which may be obtained from the Matron-in-Chief, 68 Victoria Street, S.W.

*Homes.*

(191) An elderly lady whom I know, quite destitute and dependent on relatives, is suffering from acute hysteria. Will you kindly tell me if there is any home where she would be received free of charge ?—*D. H. H.*

Write to the Hospital for Epilepsy, Paralysis, and other Nervous Disorders, 4 Maida Vale, W. If the relatives can pay anything towards her maintenance, write to the Secretary, the Holloway Sanatorium for the Insane (this includes all mental disorders), St. Ann's Heath, Virginia Water.

I should feel much obliged by the address of a home where a gentleman from India, who suffers to some extent from epilepsy and has lost his memory, could be received on very moderate terms for five months, whilst his wife is training as a monthly nurse ?—*M. A. J. M.*

The Secretary, the National Society for the Employment of Epileptics, 12 Buckingham Street, Strand, W.C., might advise you, or advertise.

I am a working man, and have a daughter who suffers from epilepsy. She has been under doctors and in hospitals without benefit. Can you tell me of a home where she could be received, or of any treatment likely to be beneficial ?—*A. T.*

See reply to *M. A. J. M.* and also apply to the Meath Home of Comfort for Epileptics, Westbrook, Godalming.

*Work.*

192. Can you tell me the best way for two nurses who wish to work together to find a district ?—*H. F.*

Advertise.

*Sea Going Nurses.*

(193) Have any definite steps been taken in regard to supplying trained nurses on ocean liners, and where should I apply to have my name added to the list of candidates ?—*S. H.*

This movement appears to have been dropped. Miss Penn, The Cottage, New Shoreham, Sussex, however, could probably give you the latest information.

*Insurance.*

(194) I am a fully-trained nurse, young and strong, yet my superintendent gives me no peace because I am not insured against sickness. I find the Royal National Pension Fund payments beyond my humble means. Can you recommend any office where sick pay is given for smaller premiums ?—*Yorkshire Girl.*

No office gives a nurse benefits equal to those conferred by the Royal National Pension Fund.

**Important Nursing Text-books.**

"The Nursing Profession: How and Where to Train." 2s. net; 2s. 4d. post free.

"The Nurses' Dictionary." By Honnor Morten. 2s. post free.

"Nursing: a Complete Text-book of Medical, Surgical, and Monthly Nursing." By Percy Lewis, M.D. 3s. 6d., post free.

"Elementary Physiology for Nurses." By C. F. Marshall, M.D. 2s. post free.

"A Complete Handbook of Midwifery for Nurses." By J. K. Watson, M.D. 6s. net; 6s. 4d. post free.

The above works are obtainable of all booksellers, or direct from The Scientific Press, Limited, 28 & 29 Southampton Street, Strand, London, W.C.

### For Reading to the Sick.

PEACE.

How oft amid the griefs of life—
Perplexed, misjudged, distressed—
O God, I waver in the strife,
And long and cry for rest.
How oft I feel—so great my need,
My courage so outworn—
As though my griefs were now indeed
Greater than could be borne.

Yet oft will come in times like these—
Come like a gracious balm—
A sense of peace, of joy, of ease,
A sense of heaven's own calm.
Ah! then my heart would fain express
What I have felt before—
'Tis not I feel my griefs are less—
I feel Thy love is more.

And some are here, O God, to-day,
Here with their voiceless grief,
O give the aid for which they pray,
O give such sweet relief,
O give Thy peace, Thy calm, Thy joys,
Here as we humbly bow.
Such Gifts nor Time nor Change destroys,
Give them, and give them *now.*

*Mackenzie Bell.*

Peace is promised to those whose hearts are stayed on God. "Thou wilt keep him in perfect peace whose mind is stayed on Thee." What then is the nature of this possession so wonderfully bestowed and preserved to us by God Himself? —Peace is that settled calm happiness which is more quiet and lasting than joy—more noble and worthy than pleasure. Pleasure may make us for a time forget pain, and a passing joy may take for a moment the place of a passing sorrow, but peace is deeper than these. Search the heart in which true peace dwells, and you will find it reaching down to the centre of life itself; pleasure, pain, joy, sorrow, may come and go, but peace abides through all.—*T. V. Fosbery.*

The peace of Christ means self-possession, the act of holding the mighty forces within us well in hand, of framing our lives on the manifest will of God, and keeping our eyes fixed on Him for direction. We read of vessels in danger of shipwreck from a storm; the crew and passengers are panic-stricken, but the captain stands on the bridge, calm and self-possessed, giving his orders, feeling sure his vessel will brave the angry sea. It is not the peace of inactivity or of freedom from pain and care: that is the peace which the world seeks to give. It may be sweet for a time, like a drug that lulls sharp pain, but it is a mockery, leaving us empty-handed and unsatisfied. The peace of Christ is the peace of a soldier who is fighting on the field of life, beneath his captain's eye, sure of victory, and who can show his hands stained and pierced with loyal struggle and his heart run through with the sword of the love of God, not closed with the dead key of love of self.

"Much peace have they, O Lord, that love Thy law." "Help me to love Thy framing of my life and to make it yield fruit for Thee. Let my mind rest in the peace and calm of the light of faith; let my heart be ruled by the dominion of love for Thee and so find peace.—*Anon.*

